# Tight Junctions and the Tumor Microenvironment

**DOI:** 10.1007/s40139-016-0106-6

**Published:** 2016-07-01

**Authors:** Ellaine Salvador, Malgorzata Burek, Carola Y. Förster

**Affiliations:** Department of Anesthesia and Critical Care, University of Wurzburg, Oberdürrbacher Straße 6, 97080 Würzburg, Germany

**Keywords:** Tight junctions, Intercellular permeability, Tumor microenvironment, Metastasis, Cancer

## Abstract

**Purpose of review:**

Tight junctions (TJs) are specialized differentiations of epithelial and endothelial cell membranes. TJs play an important role in the adhesion of cells and their interaction with each other. Most cancers originate from epithelial cells. Thus, it is of significance to examine the role of TJs in the tumor microenvironment (TME) and how they affect cancer metastasis.

**Recent findings:**

In epithelium-derived cancers, intactness of the primary tumor mass is influenced by intercellular structures as well as cell-to-cell adhesion. Irregularities of these factors may lead to tumor dissociation and subsequent metastasis. Low expression of TJs is observed among highly metastatic cancer cells.

**Summary:**

In this review, we summarized findings from current literature in consideration of the role of TJs in relation to the TME and cancer. Deeper understanding of the mechanisms leading to TJ dysregulation is needed to facilitate the design and conceptualization of new and better therapeutic strategies for cancer.

## Introduction

Since epithelial cells line hollow organs, they are prone to damage and are much exposed to carcinogens in the environment. For this reason, they demand high renewal rate. Due to their vulnerability, about 90 % of human cancers originate from the epithelial tissues [1•].

Constant remodeling of cell-to-cell contacts takes place for renewal and replacement of old or damaged cells. In addition, this process helps to incorporate newly differentiated cells without compromising the integrity of the barrier [2]. In epithelium-derived cancers, intactness of the primary tumor mass is influenced by intercellular structures as well as cell-to-cell adhesion [3]. These factors should be maintained since irregularities may lead to tumor dissociation and subsequent metastasis [4]. Tight junctions (TJs) are among those that preserve cell adhesiveness in this tumor mass. Therefore, alterations in the TJs present could result to split of the tumor mass [5]. In addition, TJs also suppress cell proliferation [6].

Owing to these facts, research has focused greatly in drawing the link between TJs and the tumor microenvironment (TME). In this review, we surveyed current literature in consideration of the role of TJs in relation to the TME and cancer.

## Characteristics of Tight Junctions

Tight junctions (TJs) are specialized differentiations of epithelial cell membranes [5]. They form a continuous intercellular barrier between epithelial cells and separate tissue spaces which regulate selective transport across the epithelium [7**•**].

TJs serve various functions. Foremost, they seal intercellular spaces and separate the apical and basolateral fluid compartments of epithelia and endothelia. They regulate epithelial and endothelial cell permeability and act as points of contact between the plasma membranes of neighboring cells, occluding the extracellular space. They also act as cell-to-cell adhesion molecules. They play a role in cell adhesion and paracellular barrier functions and form an intercellular barrier and an intramembrane diffusion fence [5]. The diffusion of solutes is regulated by TJs through size and charge selectivity and differs depending on epithelial cell type. TJs are impermeable to most macromolecules but are permeable to inorganic ions. As such, TJs, together with adherens junctions and desmosomes, maintain the integrity of the epithelial cell layer and protect multicellular organisms from the external environment [5]. In addition, they play a role in cell polarity, differentiation, growth and proliferation through their involvement in cell signaling processes. They are suggested to be involved in the regulation of cell proliferation by controlling epithelial cell microenvironment [8]. Due to this, they are able to suppress malignant phenotype of cells during tumorigenesis. Furthermore, they function as cell migration barrier. Their functions are shown to be regulated by phosphorylation. A link between barrier disruption due to TJ dysfunction and disease has long been established [9].

The main cause of lethality among cancer patients is metastasis [10]. Metastasis takes place with various prerequisites. Primarily, cancer cells need to be able to surmount the barriers, mostly epithelial and endothelial tissues consisting of cells bound together by tight junctions (TJs). Dissociated cells provide easy access to metastasizing cancer cells. Therefore, the intactness of TJs helps prevent cell dissociation [11, 12].

## Tight Junctional Components

Transmembrane proteins occludin, claudins, junctional adhesion molecules (JAMs), and tricellulin as well as intracellular scaffold proteins like zonula occludens (ZO) and cingulin comprise the molecular make-up of tight junctions (TJs).

### Transmembrane Proteins

#### Occludin

The first discovered molecular component of the TJs is occludin [13]. Although it was first suggested to form the structural unit of the TJs, it has later been found out that embryonic stem cells lacking occludin are still capable of forming TJ structures which shows that occludin is not indispensable for TJ structural assembly. To demonstrate this, occludin null mice were born without any signs of abnormal phenotype but later showed growth retardation. The TJs appear morphologically unaltered but histological abnormalities were observed in several tissues [14]. In addition, occludin knock-out mice manifest atrophic gastritis, testicular atrophy, male infertility, salivary gland dysfunction, osteoporosis, and brain calcifications [14, 15].

Apical-basal polarity is used to sense cell–cell contacts on epithelial surfaces. It has been observed that hippo pathway elements co-localize with occludin, creating a possible sensor system in pancreatic epithelial cells which may regulate their proliferation [16**•**]. It has been reported that epigenetic silencing of occludin could promote tumorigenic and metastatic properties of cancer cells [17]. For example, occludin was shown to inhibit Raf-1 signaling which induces tumor growth [18]. A low level of occludin expression results to an increased progression and metastatic potential in breast, ovarian, endometrial, and liver carcinoma [19–22]. The increased metastatic potential, however, might not be due to occludin downregulation but the activation of epithelial to mesenchymal transition (EMT) which leads to downregulation of adhesion-associated proteins [23].

#### Claudins

Of the various proteins classified as TJs, the claudin family made up of transmembrane proteins appears to be of major significance for TJ selectivity. Expression of many types of claudins is altered in cancer cells [5]. The expression of one particular claudin may be upregulated or downregulated depending on the type of cancer. Among the various members of the claudin family, claudin-1, -3, -4, and -7 are among the most frequently dysregulated both at the transcriptional and post-transcriptional levels [24]. Physiologic plasticity of the TJ involves claudin switching which is the adaptability of claudin expression and gene-specific retention in the TJ [25**•**].

Claudins are found to be involved in tumor progression and play a role in epithelial to mesenchymal transition. The expression pattern of claudins influences tumor behavior in various types of epithelial neoplasia. For example, decreased expression of claudin-1, -2, and -7 coincides with more intrusive breast carcinoma [26–29]. Meanwhile, overexpression of claudin-3 and -4 is found in several neoplasias such as in ovarian, breast, pancreatic, and prostate cancers [24]. Also, in order to clarify its role in tumor progression, the role of claudin-7 in esophageal squamous cell carcinoma was analyzed. Results proposed that the reduced expression of claudin-7 could lead to tumor progression and subsequent metastatic events [30].

#### Junctional Adhesion Molecules

Junctional adhesion molecules (JAMs) are located in cell-to-cell contacts such as tight junctions. They are expressed by leukocytes and platelets as well as by epithelial and endothelial cells [31]. They regulate cell interactions in the immune system and tight junction formation in epithelial and endothelial cells during the acquisition of cell polarity [32]. JAMs are involved in the EMT, a process that plays a vital role in the invasiveness and metastasis of various cancers. It has been shown that JAM-A upregulation could induce EMT and when JAM-A expression is silenced, EMT is reversed [33•].

### Plaque Proteins

#### Zonula Occludens

Zonula occludens (ZO) are scaffold-forming intracellular plaque proteins located between the transmembrane proteins and the actin cytoskeleton [34]. They regulate the assembly of cellular junctions. They bind actin, occludin, and claudins [35].

Several ZO are involved in cell proliferation. For instance, ZO-1 is demonstrated to interact with so-called ZO-1-associated nucleic acid binding (ZONAB) proteins, a Y-box transcription factor. This implies that ZO-1 is involved in gene expression regulation, cell proliferation, and morphogenesis of epithelial tissue [36].

Decreased expression of ZO-1 was shown to correlate with increased invasiveness in breast, colorectal, and digestive tract cancers [37]. It is also found to be involved in tumor invasion-associated EMT implicating its role in tumor growth process [38].

## Tumor Microenvironment

**Fig. 1:**
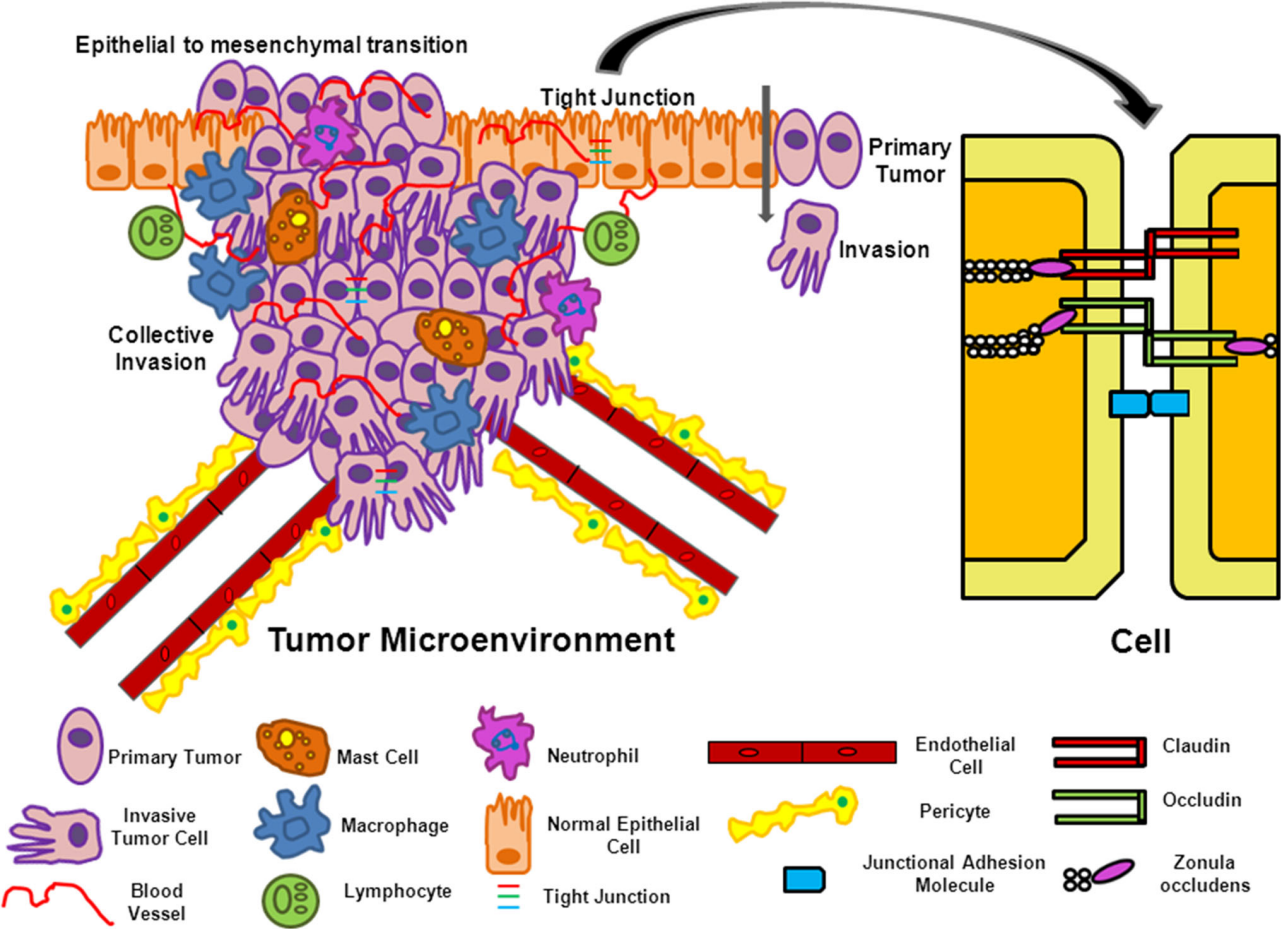
The tumor microenvironment (TME) and tight junctions (TJs). The TME is composed of normal cells, blood vessels, signaling molecules, and extracellular matrix (ECM) surrounding the tumor cells. Both malignant and nontransformed cells interacting with each other are present. Vital to this interaction are the TJs which regulate selective transport across the cells

The tumor microenvironment (TME) is composed of all the normal cells, blood vessels, signaling molecules, and extracellular matrix (ECM) surrounding the tumor cells. It is made up of malignant and nontransformed cells that interact with each other such as endothelial cells, pericytes, fibroblasts, adipocytes, and also contains cells of the immune system, the tumor vasculature, and lymphatics (Fig. 1) [39**•**].

The TME has the ability to influence tissue function as well as the development of malignancies [40]. The interaction as well as the penetration of both the endothelium and mesothelium by a tumor cell is a significant step for tumor metastasis. In order for carcinoma to invade, tumor cells need to be able to degrade the underlying basement membrane as well as the extracellular matrix (ECM) as a first step. The breakdown could then lead to invasion of neighboring tissue parenchyma [41]. However, since the TME has ways by which to maintain its normal conditions, this could only be possible once the TME is altered. In this case, deviant immune responses and altered homeostasis facilitate and modulate tumorigenesis. For instance, when the tumor acquires the capacity to bypass the means by which the TME signals it to normalcy, intercellular interactions are disrupted, forcing the TME to adapt to the growing tumor [42**•**].

Cancer cells are not the only ones responsible for the manifestation of disease. Instead, normal cell types both resident and recruited to the TME contribute to the persistence of cancer and cancer-related symptoms. For instance, alterations in both tumor and endothelial cells are essential for cancer cells to grow and metastasize. The dysregulation of significant TJ proteins leads to the loss of cell-to-cell association, cell contact inhibition that results to uncontrolled growth, and loss of adhesion to the basement as well as its degradation [43].

Low expression of TJs is observed among highly metastatic cancer cells. On the other hand, weakly metastatic tumor cells demonstrate increased TJ expression. Control of cellular proliferation involves various TJ-associated proteins such as transmembrane, adaptor and signaling proteins as well as transcription factors. When the expression levels of these proteins are changed, intercellular permeability is increased, polarity and contact inhibition are lost and expression of growth-stimulatory genes in the nucleus increases. According to research conducted on human tumors, there is a correlation of the loss of functional TJs in cancer progression and metastasis. In addition, it has also been shown that some proteins associated with TJs suppress tumors and regulate signal transduction which suggests the participation of TJ disruption in early carcinogenesis. It has been observed that mutant mice that lack specific TJ proteins develop hyperproliferative disorders [44]. The location of epithelial cells allows their direct exposure to the external environment making them prone to injury or attack by toxins, microbes, and viruses. Damage in the epithelial cells brings about loss of TJ integrity. This becomes a cue for the cells to launch a repair program involving cellular proliferative and migratory activities in the damaged area. This is accompanied by TJ reassembly in order to reform the epithelial layer. The loss of adherence between epithelial cells during the process of EMT allows them to become more motile and acquire mesenchymal characteristics. Studies show that this mechanism relates to a functional loss of E-cadherin [45]. Other events associated with EMT are loss of polarity which takes place together with the loss of TJs [46].

## Tight Junctions in Different Tumorigenic Tissues

Tight junctions (TJs) are important in the regulation of selective transport across the epithelium [7**•**]. For this reason, their involvement in cell proliferation through control of the epithelial cell microenvironment is implicated [8]. They are thus suggested to affect metastasis of tumorigenic cells. Expression of TJ-associated proteins is shown to be dysregulated in various tumorigenic tissues (Table 1).

**Table 1:** Regulation of tight junctional (TJ) proteins expression in various tumorigenic tissues

Tumor type	Tight junctions	References	Tight junctions	References
	downregulated		upregulated	
Mammary gland adenocarcinoma	Claudin 1	[63, 72•]	Claudin 3	[24, 29, 75•]
	Claudin 2	[27]	Claudin 4	[24, 29]
	Claudin 3	[72•]		
	Claudin 4	[72•]		
	Claudin 7	[28, 29, 73, 74]		
	Claudin 12	[72•]		
	ZO-1	[70, 76, 77, 78]		
	ZO-2	[70, 76, 77, 78]		
Glioma/Glioblastoma	Claudin 1	[64, 65]		
	Claudin 5	[64, 65]		
Colorectal carcinoma	Claudin 1	[86 **•**]	Claudin 2	[88]
	Claudin 4	[86•]		
	Claudin 7	[86•]		
	ZO-1	[37]		
Hepatocellular carcinoma			Claudin 1	[81]
Pulmonary carcinoma	Claudin 6	[94]		
Prostatic carcinoma			Claudin 3	[24]
			Claudin 4	[24]
Cutaneous squamous carcinoma	Claudin 1	[68, 69]	Claudin 2	[68]
	Claudin 4	[69, 70]		
	Occludin	[69]		
	ZO-1	[7•, 69]		

### Glioma and Glioblastoma

Gliomas are the most frequent tumors of the central nervous system. They are associated with a poor prognosis and high lethality. The blood–brain barrier (BBB) restricts the delivery of therapeutics into the brain making most of the systemically administered drugs ineffective in the brain tumor treatment. Progression of glioma leads to structural changes in endothelial cells of the BBB resulting in enhanced permeability and brain edema [47]. Damaged cerebrovascular endothelial cells cEND cultured in the presence or absence of astrocytic factors, for example, induced mRNA expression of inflammatory markers, alter calcium ion levels and decreased tight junction proteins claudin-5 and occludin expression [48]. Severe cerebral edema is observed in most patients with glioma and it is a main cause of mortality in glioma patients. Vasogenic edema is caused by the disturbance of the BBB either by the destruction of TJs or by the increase of endothelial fenestrations and pinocytosis [49].

Glucocorticoids (GCs) are the most common molecules used to treat tumor-associated cerebral edema [50]. GCs regulate the BBB and target occludin, claudins and VE-cadherin. Transactivation of certain target genes leads to improved barrier properties of endothelial cells [51]. For instance, at the cellular level, GCs have been shown to strengthen the BBB properties by increasing the transendothelial electrical resistance (TEER) of endothelial monolayer and decreasing the paracellular permeability for small and large molecules [52, 53]. TJs proteins occludin, claudin-5, and ZO-1 have been identified as GC targets at the BBB [52–56]. GC treatment leads to direct binding of glucocorticoid receptor to glucocorticoid response elements in the occludin promoter or to activation and binding of other transcription factors such as p54 [57, 58]. GC-mediated claudin-5 increase was observed in brain endothelial cells from different species and the induction of claudin-5 was observed at the promoter, mRNA, and protein levels [53, 54, 58]. In addition, induction of other BBB-associated claudins, claudin-1 and -12, was observed in brain endothelial cells after GC treatment [59, 60]. VE-cadherin, a component of adherens junctions, plays a role in the formation and regulation of TJs and is also induced by GC-treatment [61].

Glioma cells express high amounts of vascular endothelial growth factor (VEGF). VEGF is induced by hypoxia, promotes angiogenesis and increases BBB permeability. Increased levels of VEGF lead to down-regulation of TJ proteins claudin-5 and occludin [62]. GCs also modulate the expression of VEGF in brain tumor cells and in brain endothelial cells contributing in this way to the stabilization of the BBB [63]. Downregulation of claudin-1 and claudin-5 has been detected in human glioma and has been associated with tumor progression [64, 65]. However, the BBB in peripheral glioma remains essentially intact and this is one of the reasons for the poor treatment efficacy of this tumor [66].

### Cutaneous Squamous Carcinoma

TJs in epidermis form a barrier to prevent diffusion of molecules from and into the body. Claudin-1 knock-out mice showed fetal dehydration from skin due to impaired barrier function in the epidermis [67]. During the progression of skin tumorigenesis, changes in the expression and distribution pattern of TJ-proteins have been observed. Human cutaneous squamous carcinoma (SCC), its precursor tumors and sun-exposed skin models showed broader localization of ZO-1 and claudin-4 as well as downregulation of claudin-1 in deeper epidermal layers at the TJs in comparison to healthy skin [68–70]. In addition, SCC showed complete loss of occludin at the TJ. This feature seems to be common for different types of tumors. Occludin downregulation is associated with decreased epithelial adhesion and susceptibility to apoptosis [69]. Claudin-2 upregulation accompanied by claudin-1 downregulation was associated with tumor progression [68].

### Mammary Gland Adenocarcinoma

Cell-to-cell contacts in the epithelium are not just static points that hold the cells together since they consistently undergo remodeling and incorporation of newly differentiated cells. Although this does not lead to loss of barrier function, alterations in TJs could impact breast cancer progression due to modified cell polarity, cell fate, and cell migration. The major characteristic of cancer is abnormal proliferation. However, progression of cancer is not only determined by rapid proliferation in tumor cells. It is also due to other factors such as apoptosis resistance as well as the ability to bypass senescence pathways. In addition, individual TJ proteins may also play a role in modulating breast cancer progression.

Impairment of functional control over polarity and cell fate determination, or cell motility characteristics, may result from alterations in the TJ complex at the onset of breast cancer. Dysregulation of these cellular processes could lead to breast cancer progression and metastasis. The differentiation of the mammary gland is significant in the reduction of the risk reduction for breast cancer. In one study, a role for the estrogen receptor β (ERβ-/-) in the organization and adhesion of epithelial cells as well as for differentiated tissue morphology was suggested. Findings implicated that by facilitating terminal differentiation of the mammary gland ERβ could contribute to the risk reduction for breast cancer [71].

In another study, functional regions of occludin in human tissues and breast cancer cell lines were amplified. It has been observed that tumor tissues have truncated or variant signals of occludin. Moreover, expression of occludin in the human breast cancer cell lines tested also varied. This demonstrates the significance of occludin in TJ integrity maintenance in breast tissues [22].

One study showed a link between the expression of interleukin (IL)-18, reported to have a pro-tumor effect in various cancers, and claudins in breast cancer migration. This study showed that exogenous IL-18 could enhance breast cancer cell migration and inhibit the expression of claudin-1, 3, 4, and 12 in human breast cancer cell line MCF-7. Upon knocking down these claudins, all except claudin-1 increased breast cancer cell migration with claudin-12 generating the most effects. The results suggest that IL-18 is important for the induction of breast cancer cell migration by down-regulating claudin-12 and activating the p38 mitogen-activated protein kinase (MAPK) pathway [72•]. Another study showed that expression of claudin-7 is lower in invasive ductal carcinomas of the breast compared to normal breast epithelium [29]. In addition, reduced expression of claudin-7 corresponds to a higher tumor grade as well as metastatic disease [73]. Using immunohistochemistry and tissue microarray, it was observed that claudin-7 is strongly expressed in normal luminal epithelial cells of the breast lobule compared to ductal carcinoma and invasive breast carcinoma. Claudin-7 was significantly lower or absent in the carcinomas [74]. Still in another study conducted using two breast cancer cell lines of metastatic origin (MCF-7 and MDA-MB-415), a marked overexpression of claudin-3 protein was shown. When protein levels of claudin-3 were suppressed, the rate of cellular motility decreased. These results could indicate that claudin-3 overexpression may play an important role in the disruption of TJ integrity leading to enhanced cell motility which is a key determinant of tumor progression [75•].

Meanwhile, low ZO-1 and ZO-2 expression were observed to correlate with poor prognosis in breast cancer [70, 71, 76–78].

### Prostatic Carcinoma

Like in many organs and tissues such as the brain, the enteric epithelium or the testis where TJs abound due to the presence of a barrier where they help to regulate barrier function, it has been shown that TJs also exist in prostate tissue due to the blood-prostate barrier [72•]. In one study, the effect of hepatocyte growth factor (HGF) on TJ function in human prostate epithelial, prostate stem cell-like and prostate cancer cell lines was evaluated. It has been demonstrated that HGF could impact the metastasis of prostate cancer. During this process, TJs play a vital role and they are found to be regulated by HGF. TJ function regulation by HGF was found to be dependent on cell tumorigenicity [79•]. In another study, the overexpression of claudins is implicated in the invasive potential of human prostate cancer. The effects of flavonoids have been studied and it was observed that they subdue claudin expression which leads to suppression of cancer migration and invasion [80].

### Hepatocellular Carcinoma

Loss of TJs in the liver has been associated with malignant transformation. Nonetheless, a growing body of evidence reveals the upregulation of TJ protein expression in cancer tissue and their role in cell invasion and metastatic progression. In hepatocellular carcinoma (HCC), overexpression of claudin-1 led to increased expression of transcription factors regulating epithelial-mesenchymal transition (EMT) of human liver cells [81]. However, another study showed correlation of claudin-1 downregulation and a poor prognosis in HCC. Claudin-7 mRNA overexpression was also detected in HCC. In this case, overexpression of claudin-7 correlated with a better prognosis among patients [82]. Downregulation of claudin-5 expression in sinusoidal endothelial cells of HCC patients was correlated with a poor prognosis [83].

### Gastric/Colorectal Adenocarcinoma

Claudins are vital for the absorption of nutrients in the small intestine [84]. In the same way, they are vital in cell proliferation and transformation during cancer. For instance, in colon cancer, claudin-1 was shown to promote transformation as well as metastatic behavior [85]. Expression of claudin-1, -4, and -7 was found to decrease in colorectal cancer. This implies critical effects on cell proliferation, motility, invasion, and immune response against the tumor [86**•**]. One study did an analysis of the allele frequencies on three common single nucleotide polymorphisms (SNPs) in the genes for claudin-1 and 7 in colon cancer patients. It was observed that polymorphisms in both claudins investigated are related to differentiation and tumor state in colon cancer [87**•**]. On the other hand, deficiency in claudin-15 results to megaintestine and a decreased paracellular ion permeability of the intestinal epithelium. Nonetheless, no tumorigenesis was detected among those exhibiting the phenotype [8]. Also, in colorectal cancer, it has been demonstrated that resveratrol, a naturally occurring polyphenol, upregulates intercellular junctions such as desmosomes, gap- and tight junctions (claudin-2), and adhesion molecules (E-cadherin). On the other hand, it downregulates the NF-κB pathway. These processes lead to inhibition of the EMT phenotype [88]. In another study, CITED4, a transcriptional cofactor deregulated in colorectal cancer, was knocked down via shRNA-mediation in the colorectal cancer cell line SW480. Changes in proliferation, apoptosis/cell cycle, migration, invasion, colony formation, and adhesion were analyzed. Decreased cellular proliferation and modulation of actin-associated adherens junctions/TJs expression have been observed [89].

Meanwhile, the function of junctional adhesion molecules (JAMs) which comprise the integral parts of TJs in the gastric epithelium and in gastric cancer cell proliferation, invasion, and apoptosis was investigated. It has been shown that JAM-A promotes the proliferation but inhibits apoptosis of gastric cancer [90]. On the other hand, decreased ZO-1 expression was noted in the human digestive tract.

### Pulmonary Carcinoma

The expression of TJ proteins such as claudins in different lung tumors varies. For instance, occludin was found in adenocarcinomas but not in squamous cell carcinomas, small or large cell carcinomas [91]. Meanwhile, claudins show variable patterns of expression in tumor cells. Squamous cell carcinomas express claudin 1 but not claudin 5 while adenocarcinomas express claudin 5 but not claudin 1 [20]. Epithelial metastases of lung tumors showed a 50–70 % expression of claudins 1, 2, 3, 4, and 5 and a 90 % expression of claudin 7 [92]. TJ proteins are usually overexpressed in lung tumors. Besides TJ protein overexpression, matrix metalloproteinases are also increased which leads to spread of the tumor [93]. On the other hand, immunohistochemical study of tissue from patients with nonsmall cell lung cancer revealed low claudin-6 expression indicative of a worse prognosis [94]. Nonetheless, permeability of pulmonary epithelium can be governed by various factors. For example, in using human alveolar epithelium cell line H441, it has been shown that soluble factors obtained from human lung endothelial cell line HPMEC-ST1.6R could influence the barrier properties of the former [95]. Thus, in the TME where there is interplay of different cell types, the possible effects that the different interactions among these cells bring about need to be further explored.

## Cytokines, Tight Junctions, and the Tumor Microenvironment

Dysfunction in the TME as well as the epithelium can be crucial for carcinogenesis [96]. It is suggested that cancer cases are triggered by mutation and inflammation, although most cases have unknown origin [97]. Chronic inflammation could lead to cellular events that promote cell transformation resulting to cancer formation. Inflammation activates cytokine production within the TME [98]. There is greater oxidative stress in the microenvironment surrounding inflammation compared to normal. Inflammation leads to expression of cytokines which in turn activates the inflammatory cascade [97]. Moreover, cytokines contribute to the promotion of cell tumor proliferation as well as apoptosis inhibition and anti-tumor immunity suppression. When epithelium surrounding the stroma is altered, it also alters the stroma along with the mediators leading to TME formation [99]. In the same manner, when TJs within the TME are disrupted, cytokine-mediated perturbation of TJs occurs which in turn leads to increased paracellular permeability [100] and promotes TJ remodeling [101].

## Conclusions

The tumor microenvironment (TME) engages a complex interaction among the component cells that comprise it. Communication and cross-talk in the TME are maintained by the tight junctions (TJs). Dysregulation of TJs at the TME could affect cell permeability which could influence tumor metastasis. Hereof, deeper understanding of the mechanisms surrounding TJ dysregulation is needed to facilitate the design and conceptualization of new and better therapeutic strategies for cancer.
